# Toxic Effects of Carbaryl Exposure on Juvenile Asian Seabass (*Lates calcarifer*)

**DOI:** 10.3390/jox14030051

**Published:** 2024-07-10

**Authors:** Junhua Huang, Zhengyi Fu, Wei Yu, Zemin Bai, Zhenhua Ma

**Affiliations:** 1 Key Laboratory of Efficient Utilization and Processing of Marine Fishery Resources of Hainan Province, Sanya Tropical Fisheries Research Institute, Sanya 572018, China; 2 South China Sea Fisheries Research Institute, Chinese Academy of Fishery Sciences, Guangzhou 510300, China; 3 Tropical Aquaculture Research and Development Center, South China Sea Fisheries Research Institute, Chinese Academy of Fishery Sciences, Sanya 572018, China; 4 Hainan Engineering Research Center for Deep-Sea Aquaculture and Processing, Sanya 572018, China; 5 International Joint Research Center for Conservation and Application of Fishery Resources in the South China Sea, Sanya 572018, China; 6 College of Science and Engineering, Flinders University, Adelaide 5001, Australia; 7 Yazhou Bay Agriculture and Aquaculture Co., Ltd., Sanya 572025, China

**Keywords:** antioxidant enzyme activity, serum biochemical parameters, histopathology, immune gene expression

## Abstract

This study examines the physiological and immunological effects of 0.5 ppm carbaryl exposure on juvenile Asian seabass (*Lates calcarifer*) over 12 h to 72 h. Notable results include decreased activities of liver enzymes catalase (CAT), lactate dehydrogenase (LDH), and glutathione peroxidase (GSH-PX), while superoxide dismutase (SOD) levels remained stable, with the lowest activities of CAT and GSH-PX observed at 72 h. Serum biochemistry revealed increased alkaline phosphatase (AKP) and acid phosphatase (ACP) at 24 h, with declining aspartate aminotransferase (AST) and a peak in creatinine at 48 h. Histopathological analysis showed carbaryl-induced necrosis in liver and spleen cells, and increased melanomacrophage centers in both organs. Additionally, immune gene expression analysis indicated an upregulation of heat shock proteins and consistent elevation of complement component C3 and interleukin-8 (IL-8). These findings suggest that carbaryl exposure significantly impairs organ function and modulates immune responses in *L. calcarifer*, underlining the need for further research on protective strategies against pesticide impacts in aquaculture.

## 1. Introduction

The Asian seabass (*Lates calcarifer*), widely distributed in the Asia–Pacific region, is one of the most important finfish in the marine aquaculture industry in Australia and Asian countries [1]. Owing to its substantial nutritional value and swift growth rate, the Asian seabass has emerged as a principal species within the aquaculture sector of Southern China [2]. Carbaryl, a globally renowned carbamate insecticide known for its exceptional pest control efficacy across various crop types, is extensively used in the coastal agricultural regions of Southern China [3]. This pesticide plays an essential role in both increasing agricultural productivity and ensuring food security [4]. However, the extensive use of pesticides carries significant downsides, as they can potentially harm local estuarine and coastal aquaculture through the discharge of agricultural wastewater [5,6].

The liver plays a vital role in several key metabolic functions [7] and is the primary organ for the accumulation, biotransformation, and excretion of pollutants in fish, including the degradation and bioactivation of pesticides [8,9]. In experimental studies, assessing the biochemical and histological changes in fish livers has become an important tool for monitoring environmental exposure to pollutants [10]. Exposure to pollutants in aquatic ecosystems can enhance the formation of reactive oxygen species within cells, leading to oxidative damage to biological systems [11]. Studies have shown that Nile tilapia (*Oreochromis niloticus*) exposed to 0.5 ppm carbaryl exhibited a sublethal condition, with a few liver cells showing necrosis and a decreasing trend in enzymes like superoxide dismutase, catalase, and glutathione peroxidase [12]. Boran et al. (2010) identified focal necrosis in the liver, head kidney, and spleen of rainbow trout (*Oncorhynchus mykiss*) during static tests involving exposure to carbaryl [13]. Furthermore, carbaryl can impair the normal neurological functions of trout by inhibiting acetylcholinesterase, leading to a loss of normal neural behavior and reduced predation efficiency [14]. Watershed research has consistently shown that carbaryl poses significant risks due to its acute toxicity to various small marine fish species as well as to aquatic and benthic invertebrates in estuarine environments [15]. Numerous studies have documented these effects, illustrating how carbaryl exposure can disrupt local ecosystems and affect the survival of marine life [16]. For instance, Arunachalam and Palanichamy (1982) and Dumbauld, Brooks, and Posey (2001) highlighted the vulnerability of these organisms when exposed to pesticides, with outcomes ranging from altered behavior and physiological damage to increased mortality rates. This body of research underscores the need for stricter regulations and improved management practices to mitigate the environmental impact of pesticide use in these sensitive environments [17,18].

The immune system in fish serves as the principal defense against environmental pathogens and toxicants [19]. Pesticides interact with intracellular receptors and signaling molecules in fish, thereby modulating gene expression regulatory mechanisms. This interaction precipitates notable alterations in the expression of genes associated with immune, metabolic, and neurological functions, consequently disrupting normal physiological and immune responses in these organisms [20]. Evidence shows that exposure to the pesticide carbaryl induces an upregulation in immune gene expression in hybrid catfish [21]. Similarly, exposure to the organochlorine pesticide MXC elevates VTG gene expression in male largemouth bass (*Micropterus salmoides*), with VTG possessing antioxidant properties that shield the host from oxidative stress and pose potential risks to the endocrine system [22,23]. These alterations in immune gene expression not only provide insights into the immune status of the fish but also elucidate their molecular response to chemical pollutants [24].

Based on environmental data surveys, carbaryl concentrations in the western part of Japan’s Seto Inland Sea range from 4.3 to 0.21 µg/L, typically staying below 0.5 ppm in natural settings [25]. Due to limited data on carbaryl’s impact on fish, this study employed Asian seabass (*L. calcarifer*) as a model organism. The literature indicates that juvenile *Dicentrarchus labrax* begin to die after 24 h of exposure to 0.5 ppm carbaryl [26]. Considering the possible sublethal presence of carbaryl in natural waters, this experimental design involved exposing juvenile *L. calcarifer* to 0.5 ppm of carbaryl for a short duration to explore its potential impacts on fish health more comprehensively.

The experimental protocol included periodic assessments of antioxidant enzyme activities in juvenile Asian sea bass to evaluate the repercussions of carbaryl exposure on their antioxidant defense systems. Further, this study conducted histological analyses of immune organs and quantified expressions of related immune genes, thereby uncovering the impacts of carbaryl on the immune functionality of *L. calcarifer*. These impacts encompassed potential organ damage and alterations in immune responsiveness. In recent years, the widespread use of carbaryl pesticides in agriculture has raised significant concerns regarding their environmental impact. Previous studies have indicated that these pesticides possess potential toxicity in aquatic environments, posing a threat to marine life. This study aims to comprehensively investigate the effects of carbaryl on fish health, including disruptions in enzymatic activity, potential organ damage, and compromised immune functions. By thoroughly analyzing these impacts, this research will provide crucial insights into the detrimental effects of pesticide contamination on aquatic organisms and offer scientific support for the development of more effective management and protection strategies.

## 2. Materials and Methods

### 2.1. Experimental Design and Sample Preparation

Juvenile *L. calcarifer*, with an average weight of 88.66 ± 9.17 g, were procured from the Tropical Aquaculture Research and Development Center at the South China Sea Fisheries Research Institute, Hainan, China. The specimens were uniformly distributed into two groups, with each group comprising three replicates and each replicate containing 15 individuals housed in 1000 L tanks. Throughout a seven-day acclimatization period, the water quality was meticulously controlled within stringent parameters (salinity: 32 ± 0.50 psu; temperature: 27 ± 0.50 °C; pH: 8.10 ± 0.15; dissolved oxygen: >6.50 mg·L^−1^; ammonia nitrogen: <0.1 mg·L^−1^; nitrite nitrogen: <0.02 mg·L^−1^) under ambient light conditions. Feeding was conducted bi-daily at 08:00 and 17:00 using commercial pellets supplied by Sanyou Middle-Part Feed (Weifang, China)

The experimental design included a control group (CG) with 0 ppm carbaryl, and an experimental group (EG) exposed to 0.5 ppm carbaryl, with three replicates for each condition. The CG served as a natural seawater baseline, whereas the EG was designed to mimic the potential carbaryl contamination under natural environmental conditions [24]. carbaryl powder (97% purity, Aladdin Reagent, Shanghai, China) was administered to the EG to achieve the desired concentration, ensuring complete solubilization in the aquaculture medium.

At 12, 24, 48, and 72 h after administering carbaryl, three fish from each tank were randomly selected, anesthetized with a high dose of MS-222, and quickly dissected to collect the liver, head kidney, spleen, and blood from the caudal vein. Collected tissues were immediately plunged into liquid nitrogen for rapid freezing followed by storage at −80 °C for subsequent analyses of enzyme activity and RNA extraction.

### 2.2. Liver Enzyme Activity Determination and Serum Biochemical Index Analysis

Approximately 0.1 g of liver tissue was collected from each fish and placed into a 2 mL centrifuge tube. This was followed by the addition of a ninefold volume of 0.86% saline solution. The tissue was homogenized on ice using a Prima PB100 handheld homogenizer (Gloucester, England). The homogenate was centrifuged at 3500× *g* for 10 min, and the supernatant was collected and stored at −80 °C for enzyme activity analysis.

Blood samples were retained in 2 mL centrifuge tubes at room temperature for 1 h, followed by centrifugation at 3000 rpm and 4 °C for 10 min using a high-speed refrigerated centrifuge (Gloucester, Prima PB100, England). The samples were stored at −20 °C until analysis.

Activities of total superoxide dismutase (SOD), lactate dehydrogenase (LDH), catalase (CAT), glutathione peroxidase (GSH-PX), along with serum creatinine (CRE) content, serum alkaline phosphatase (AKP), serum acid phosphatase (ACP), serum glutamate oxaloacetate transaminase (AST), and malondialdehyde (MDA) were determined using commercial diagnostic kits (Nanjing Jiancheng Bioengineering Institute, Nanjing, China) following the manufacturer’s instructions. The protein content in the homogenate was measured using a total protein quantification kit (Nanjing Jiancheng Bioengineering Institute, Nanjing, China).

### 2.3. Histological Analysis

Freshly harvested liver, head kidney, and spleen tissues were fixed using 4% paraformaldehyde. The fixed samples were then embedded in paraffin blocks, and serial cross-sections (4 µm thick) were prepared using a Leica RM 2016 rotary microtome (Leica Instruments GmbH, Shanghai, China). General histological analysis was conducted using hematoxylin and eosin (H&E) staining. Each tissue section mounted on a slide was permanently sealed with neutral resin. Observations of the sections were made under a Nikon Eclipse Ni-U upright microscope (Nikon Instruments Inc., Tokyo, Japan) at 400× magnification. Pathological alterations in the liver, kidney, and spleen were examined in ten randomly selected sections from each fish.

### 2.4. Validation of RNA-Seq Results by Quantitative Real-Time PCR (qRT-PCR)

RNA was extracted from liver tissue samples. Total RNA was extracted using TRIzol reagent (Invitrogen, Carlsbad, CA, USA) according to the manufacturer’s instructions. The integrity, concentration, and purity of the RNA were assessed using agarose gel electrophoresis, Nanodrop 2000 (Thermo Fisher Scientific, Waltham, MA, USA), and the 2100 Bioanalyzer (Agilent Technologies, Waldbronn, Germany). Only high-quality RNA samples (OD260/280 = 1.8 to 2.2, OD260/230 ≥ 2.0, 28S:18S ≥ 1.0, total RNA > 10 μg) were used for subsequent experiments. A volume of 2 μL of total RNA was transferred using a micropipette. Reverse transcription was then performed using the EasyScript All-in-One First-Strand cDNA Synthesis SuperMix for qPCR (One-Step gDNA Removal), following the kit’s protocol. This synthesized the first strand of cDNA, which was then used as a template for downstream gene expression analysis.

In the quantification of immune gene expression at various time points, we used the gene expression levels of the control group at 12 h as a normalized reference for relative quantification of gene expression. To validate the reliability of our RNA-Seq results, we selected four immune-related genes and verified them using quantitative real-time PCR (qRT-PCR) on a real-time qPCR analysis system (Analytik Jena GmbH, Jena, Germany) with SYBR Green (Tiangen Biotech Co., Ltd., Beijing, China). Specific primers were designed using Primer Premier 5 software (Table 1). The reaction mixture (20 μL) comprised 10 μL of 2× RealUniversal PreMix, 0.6 μL of each primer (10 μM), and 2 μL of diluted cDNA. The samples were initially denatured at 95 °C for 15 min, followed by 40 cycles of amplification (95 °C for 10 s, 58 °C for 20 s, 72 °C for 30 s). A melting curve analysis was performed at the end of each qRT-PCR cycle to ensure the specificity of the products and the absence of primer dimers. Each experiment included a no-template control to confirm that the PCR reaction mixture was free of contamination. Relative mRNA expression levels of the target genes were quantified using the 2^−ΔΔCt^ method, with β-actin as the internal reference gene. The reaction efficiency was between 90 and 110%, and the Pearson correlation coefficient (R^2^) was >0.97.

### 2.5. Calculations and Statistical Analysis

In this study, data organization was conducted using Excel 2021 software. The creation of graphs was carried out using the Origin 2021 software, while statistical analysis, including tests for significant differences, was performed using SPSS 26.0 software. Specifically, we employed independent *T*-tests to compare data across different groups. The significance level for all statistical tests was set at *p* < 0.05.

## 3. Results

### 3.1. The Impact of Carbaryl Exposure on the Liver of L. calcarifer

The two-way ANOVA analysis demonstrated that the effects of carbaryl pesticide exposure and exposure duration on the enzymatic activities in the liver of juvenile *L. Calcarifer* vary significantly. Notably, CAT enzyme activity showed significant variations due to the combined effects of carbaryl exposure and exposure duration, as documented in Table S1 (*p* = 0.001). In contrast, the interaction effects of carbaryl stress and exposure duration on LDH and GSH-PX activities were not statistically significant, as indicated in Tables S2 and S3 (*p* = 0.796 and *p* = 0.238, respectively). Similarly, the interaction between carbaryl exposure and duration of exposure did not significantly affect changes in SOD activity, which is detailed in Table S4 (*p* = 0.961).

**Figure 1:**
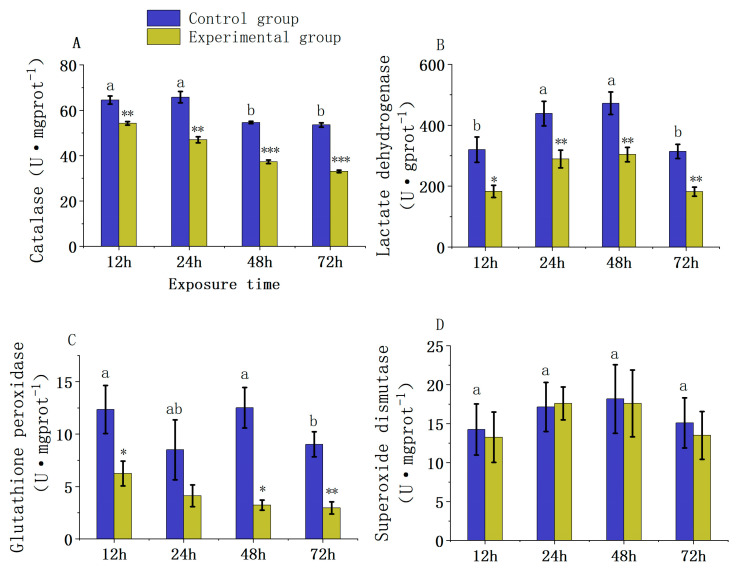
Changes in antioxidant enzyme activities in the liver of *L. calcarifer* juveniles exposed to 0.5 ppm carbaryl ((**A**): Catalase (CAT) activity; (**B**): Lactate Dehydrogenase (LDH) activity; (**C**): glutathione peroxidase (GSH-PX) activity; (**D**): superoxide dismutase (SOD) activity. Symbols in the figure represent statistical significance: “*” for *p* < 0.05, “**” for *p* < 0.01, and “***” for *p* < 0.001). Different letters indicate statistically significant differences.

Under the stress conditions of a 0.5 ppm carbaryl environment, the activity of CAT in the liver of juvenile *L. calcarifer* (Figure 1A) gradually decreased with increasing exposure time to carbaryl. Furthermore, the average CAT activity in the experimental groups at 12, 24, 48, and 72 h was significantly lower than that in the control group by 1.19-fold, 1.4-fold, 1.47-fold, and 1.62-fold, respectively (12 h: *p* = 0.001; 24 h: *p* = 0.003; 48 h: *p* = 0.001; 72 h: *p* = 0.001). In the control group, CAT activity at 48 h and 72 h was significantly lower than at 12 h and 24 h, showing a 1.2-fold decrease (*p* = 0.007). 

The activity of LDH in the liver (Figure 1B) increased at 12, 24, and 48 h of exposure to carbaryl, but decreased at 72 h, with LDH activity in the experimental groups at 12, 24, 48, and 72 h being significantly lower than that in the control group by factors of 1.75-fold, 1.52-fold, 1.55-fold, and 1.72-fold, respectively (12 h: *p* = 0.016; 24 h: *p* = 0.008; 48 h: *p* = 0.005; 72 h: *p* = 0.002). In the control group, LDH activity at 24 h and 48 h was significantly higher than at 12 h and 72 h, exhibiting a 1.4-fold increase (*p* = 0.036). As carbaryl exposure time increased under stress conditions, the activity of GSH-PX in the liver of juvenile *L. calcarifer* (Figure 1C) progressively declined, with significant reductions at 12, 48, and 72 h compared to the control group of 1.98-fold, 3.88-fold, and 3.06-fold, respectively (12 h: *p* = 0.027; 48 h: *p* = 0.011; 72 h: *p* = 0.005). At 24 h, the difference in GSH-PX activity between the experimental and control groups was not significant (24 h: *p* = 0.100). In the control group, GSH-PX activity at 24 h was lower than at 12 h and 48 h, although the difference was not statistically significant. However, GSH-PX activity at 72 h was significantly reduced by approximately 1.36-fold compared to both 12 h and 48 h (*p* = 0.047). Meanwhile, the activity of SOD in the liver (Figure 1D) initially increased and then decreased as exposure time extended, but there were no significant differences in SOD activity between the experimental and control groups at any time point (12 h: *p* = 0.730; 24 h: *p* = 0.850; 48 h: *p* = 0.880; 72 h: *p* = 0.570).

### 3.2. The Effects of Carbaryl Exposure on Serum Biochemical Indices in L. calcarifer

According to a two-way ANOVA, the exposure to carbaryl pesticide and the duration of exposure significantly affect the serum biochemical markers in juvenile *L. Calcarifer*. Specifically, the interactions between carbaryl stress and exposure duration significantly influence the activities of AKP and ACP, as shown in Tables S5 and S6 (*p* = 0.005 and *p* = 0.009, respectively). Similarly, serum creatinine levels are markedly affected by these interactions, detailed in Table S9 (*p* = 0.001). In contrast, the activities of serum MDA and AST show no significant changes attributable to the interaction of carbaryl exposure and exposure duration, as indicated in Tables S7 and S8 (*p* = 0.915 and *p* = 0.057, respectively).

**Figure 2:**
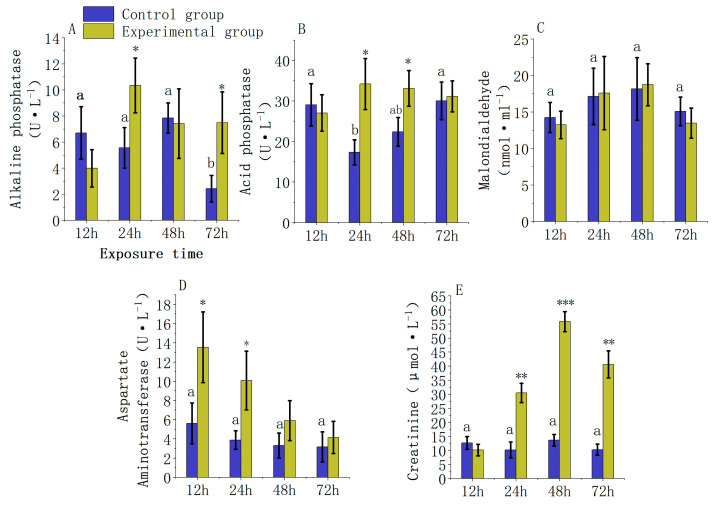
Changes in serum biochemical parameters of *L. calcarifer* juveniles exposed to 0.5 ppm carbaryl ((**A**): alkaline phosphatase (AKP) activity; (**B**): acid phosphatase (ACP) activity; (**C**): malondialdehyde (MDA); (**D**): aspartate aminotransferase (AST) activity; (**E**): creatinine. Symbols in the figure represent statistical significance: “*” for *p* <0.05, “**” for *p* <0.01, and “***” for *p* < 0.001). Different letters indicate statistically significant differences.

Under the stress conditions induced by 0.5 ppm carbaryl, the activity of AKP in the serum of juvenile *L. calcarifer* (Figure 2A) showed no significant differences between the experimental and control groups at 12 and 48 h (12 h: *p* = 0.14 and 48 h: *p* = 0.82). At 24 and 72 h, AKP activity was significantly higher than in the control group by 1.85-fold and 3.08-fold, respectively (24 h: *p* = 0.038 and 72 h: *p* = 0.049). Within the trends observed in the control group, alkaline phosphatase (AKP) activity at 72 h was significantly lower than at 12 h, 24 h, and 48 h, approximately by a factor of 2.8 (*p* = 0.02). With increasing exposure time to carbaryl, the activity of ACP in the serum (Figure 2B) initially increased and then gradually decreased. At 24 h and 48 h, ACP activity in the experimental group was significantly higher than in the control group by 1.97-fold and 1.48-fold, respectively (24 h: *p* = 0.027 and 48 h: *p* = 0.032), while at 12 and 72 h, the differences were not significant (12 h: *p* = 0.643 and 72 h: *p* = 0.768). Within the observed trends in the control group, acid phosphatase (ACP) activity at 24 h was significantly lower than at 12 h and 72 h, by approximately 1.73-fold (*p* = 0.040).

Additionally, the content of MDA in the serum (Figure 2C) increased initially and then decreased with longer exposure times to carbaryl, with no significant differences found between the experimental and control groups at any time point (12 h: *p* = 0.570; 24 h: *p* = 0.910; 48 h: *p* = 0.860; 72 h: *p* = 0.380). The activity of AST in the serum (Figure 2D) gradually decreased with increased exposure time. At 12 h and 24 h, AST activity in the experimental group was significantly higher than in the control group by 2.41-fold and 2.59-fold, respectively (12 h: *p* = 0.044 and 24 h: *p* = 0.048), but there were no significant differences at 48 and 72 h (48 h: *p* = 0.150 and 72 h: *p* = 0.490). Lastly, the creatinine content in the serum (Figure 2E) initially increased and then decreased over time; at 12 h, the creatinine levels were lower in the experimental group compared to the control group, though the difference was not significant (12 h: *p* = 0.220). At 24 h, 48 h, and 72 h, the levels were significantly higher than those in the control group by 2.98-fold, 4.09-fold, and 3.94-fold, respectively (24 h: *p* = 0.002; 48 h: *p* = 0.001; 72 h: *p* = 0.004).

### 3.3. Histological Analysis

**Figure 3:**
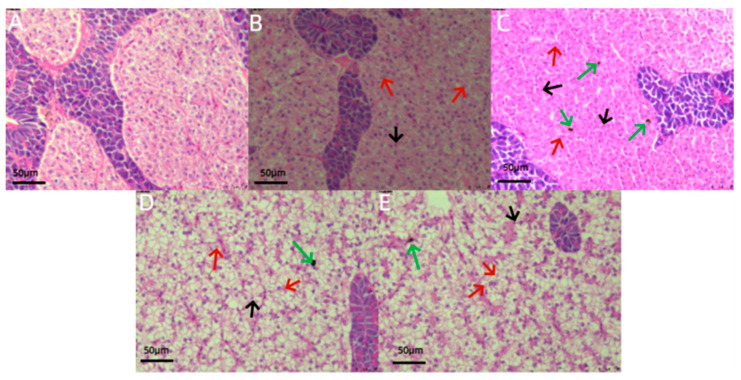
Histological sections of the liver tissue in juvenile *L. calcarifer* exposed to 0.5 ppm carbaryl. (**A**): Control group; (**B**): experimental group at 12 h; (**C**): experimental group at 24 h; (**D**): experimental group at 48 h; (**E**): experimental group at 72 h (red arrow: hepatocyte swelling; green arrow: hepatocyte necrosis; black arrow: nuclear shrinkage; scale bar: 50 μm).

**Figure 4:**
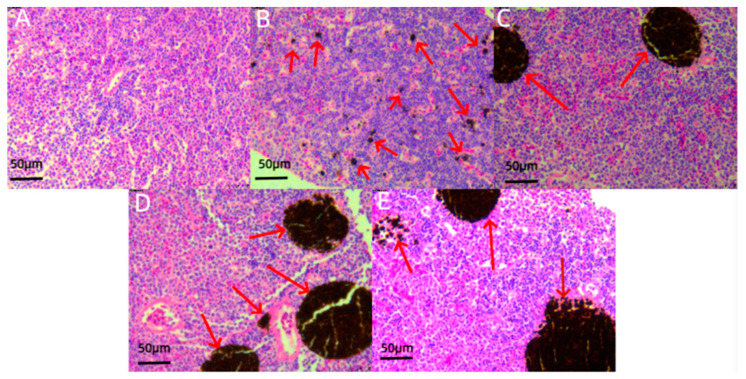
Histological sections of the head kidney tissue in juvenile *L. calcarifer* exposed to 0.5 ppm carbaryl. (**A**): Control group; (**B**): experimental group at 12 h; (**C**): experimental group at 24 h; (**D**): experimental group at 48 h; (**E**): experimental group at 72 h; (red arrow: melanomacrophage centers, MMCs; scale bar: 50 μm).

**Figure 5:**
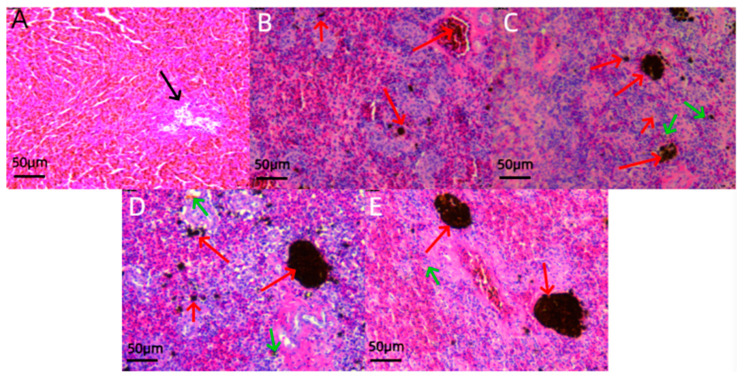
Histological sections of the spleen tissue in juvenile *L. calcarifer* exposed to 0.5 ppm carbaryl. (**A**): Control group; (**B**): experimental group at 12 h; (**C**): experimental group at 24 h; (**D**): experimental group at 48 h; (**E**): experimental group at 72 h; (red arrow: melanomacrophage centers, MMCs; black arrow: lymphocytes; green arrow: necrotic spleen cells; scale bar: 50 μm).

Histological analysis of liver tissue sections (Figure 3) indicated significant edema and necrosis in hepatocytes of juvenile *L. calcarifer* when exposed to 0.5 ppm carbaryl. Particularly, after exposure durations of 48 and 72 h, severe cellular necrosis and edema were observed, along with a few cases of hepatocyte nuclear shrinkage. Additionally, examination of head kidney tissue sections (Figure 4) revealed an increasing trend in the number of melanomacrophages (MMCs) with prolonged exposure to carbaryl. Notably, the highest number of MMCs was observed after 72 h of exposure in the experimental group. Further analysis of spleen tissue sections (Figure 5) under the influence of carbaryl showed that individual spleen cells exhibited necrosis, and there was a significant increase in MMCs in the experimental groups. Specifically, the number of MMCs peaked after 48 h of exposure.

### 3.4. Analysis of Immune Gene Expression Levels

**Figure 6:**
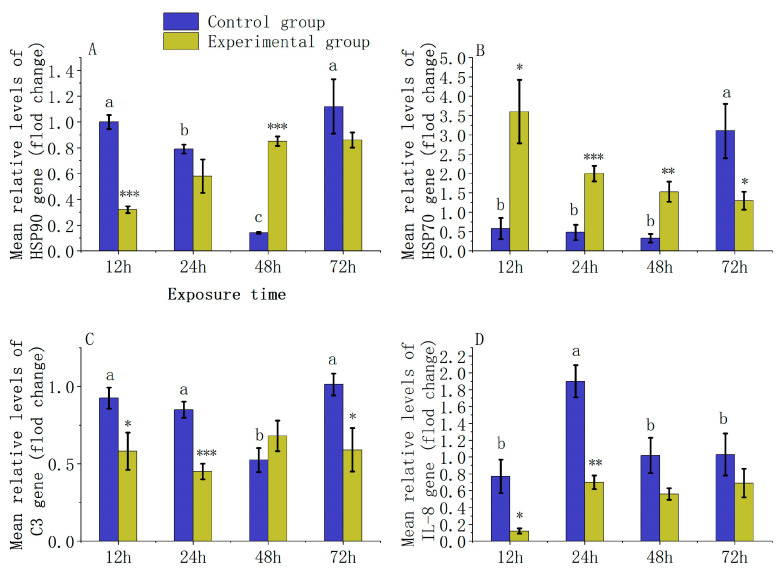
Immune genes in the liver of juvenile *L. calcarifer* were expressed following exposure to 0.5 ppm carbaryl. ((**A**): HSP90 gene; (**B**): HSP70 gene; (**C**): Complement C3 gene; (**D**): IL-8 gene. Symbols in the figure represent statistical significance: “*” for *p* < 0.05, “**” for *p* < 0.01, and “***” for *p* < 0.001). Different letters indicate statistically significant differences.

Under the environmental stress of 0.5 ppm carbaryl, juvenile *L. calcarifer* exhibited a gradual increase in the expression of the HSP90 gene (Figure 6A) with prolonged exposure to carbaryl. At 12 h, the expression levels in the experimental group were significantly lower than those in the control group by 3.12-fold (12 h: *p* = 0.001) and at 48 h, significantly higher than the control group by 6.07-fold (48 h: *p* = 0.001). At 24 and 72 h, the experimental group showed lower expression levels compared to the control group, but the differences were not statistically significant (24 h: *p* = 0.120 and 72 h: *p* = 0.160). Conversely, the expression of the HSP70 gene in juvenile *L. calcarifer* (Figure 6B) showed a gradual decreasing trend as the exposure time to carbaryl increased. At 12, 24, and 48 h, the expression levels in the experimental group were significantly higher than those in the control group by 6.2-fold, 4.17-fold, and 4.63-fold, respectively (12 h: *p* = 0.016; 24 h: *p* = 0.001 and 48 h: *p* = 0.007), but were significantly lower by 2.38-fold at 72 h (72 h: *p* = 0.034). Regarding the C3 gene expression in juvenile *L. calcarifer* exposed to carbaryl (Figure 6C), slight fluctuations were observed in the experimental group. At 48 h, the C3 gene expression was slightly higher than that in the control group, though the difference was not significant (48 h: *p* = 0.100). At 12, 24, and 72 h, the expression levels in the experimental group were significantly lower than those in the control group by 1.59-fold, 1.88-fold, and 1.71-fold, respectively (12 h: *p* = 0.023; 24 h: *p* = 0.001 and 72 h: *p* = 0.022). In terms of IL-8 gene expression (Figure 6D), juvenile *L. calcarifer* in the experimental group showed an increase at 24 h following exposure to carbaryl, followed by minor fluctuations; however, levels remained lower than those in the control group throughout. At 12 and 24 h, the levels in the experimental group were significantly lower than those in the control group by 6.42-fold and 2.71-fold, respectively (12 h: *p* = 0.028 and 24 h: *p* = 0.003), but not at 48 and 72 h (48 h: *p* = 0.052 and 72 h: *p* = 0.130).

## 4. Discussion

### 4.1. Effect of Chemical Pesticide Exposure on Liver Antioxidant Enzyme Activity in Fish

The liver in fish serves as the primary detoxification organ, tasked with neutralizing and clearing exogenous compounds [27]. Under the stress of exposure to the pesticide carbaryl, antioxidant enzymes such as SOD, GSH-Px, and CAT play a crucial role. These enzymes help defend against and neutralize reactive oxygen species (ROS) produced during metabolic activities. Excessive accumulation of ROS (e.g., H_2_O_2_, -OH, O^2−^) can be highly toxic to cells, but animals have an antioxidant system designed to mitigate these negative effects [28]. SOD, GSH-PX, and CAT are important protective enzymes that are essential in scavenging free radicals, preventing oxidative damage within cells, and maintaining redox balance [29]. Studies have shown that in juvenile rainbow trout (*Oncorhynchus mykiss*), CAT activity in the liver significantly decreases under exposure to 1 mg·L^−1^ carbaryl for 48 h, with a general declining trend in other antioxidant enzyme activities (CbE and GST) as well [30]. Additional research indicates that carbaryl exposure can inhibit the activity of antioxidant enzymes in the liver of fish [31,32]. Similarly, this study found that CAT and GSH-PX activities also declined in the liver of juvenile *L. calcarifer* under 0.5 ppm (mg·L^−1^) carbaryl exposure, suggesting that carbaryl may inhibit antioxidant enzyme activity, potentially causing liver damage in fish. Compared to the overall trend observed in the control group, CAT activity at 48 h and 72 h was significantly lower than at 12 h and 24 h, and GSH-PX activity at 72 h was significantly lower than at 12 h and 48 h. This suggests that the expression and activity of these enzymes may be regulated by the processes of protein synthesis and degradation. Over time, there could be an increase in protein degradation activities or a slowdown in the rate of new protein synthesis. Unlike other cases, SOD activity did not differ significantly from the control group in this experimental setup. This could be due to the regulatory effects of other antioxidant components in the fish (such as glutathione and vitamin E), which might provide SOD with greater stability to maintain cellular redox balance [33].

Lactate dehydrogenase (LDH) is a crucial enzyme involved in the redox reactions between lactate and pyruvate during glycolysis and gluconeogenesis. A reduction in LDH activity is commonly associated with chemical drug stimulation, liver diseases, blood disorders, and cellular aging [34]. Studies have indicated that chemical pesticides can inhibit LDH activity in organisms, leading to liver damage [35]. In this study, we observed a significant reduction in LDH activity in the liver of juvenile *L. calcarifer* when exposed to carbaryl, compared to the control group. This finding is consistent with previously mentioned results and indicates that carbaryl exposure may initially suppress LDH activity. However, paradoxically, this suppression could be indicative of carbaryl’s potential to eventually elevate LDH activity as part of a stress response in liver tissues. Such an increase in LDH activity might contribute to liver damage and potentially accelerate the aging of hepatic cells. The exact mechanisms by which carbaryl influences LDH activity warrant further investigation, as they may provide critical insights into how environmental stressors affect aquatic organisms at the cellular level. Relative to changes within the control group, activity levels at 24 h and 48 h were significantly higher than at 12 h and 72 h. This could be attributed to hepatocytes reaching a peak in physiological activity within a certain experimental period. The peak physiological activity of hepatocytes in the control group at 24 h and 48 h may reflect an optimal state of cellular metabolic activity, exhibiting higher activity levels.

### 4.2. Effect of Chemical Pesticide Exposure on Serum Biochemical Indexes in Fish

Serum biochemical markers in fish are crucial tools for assessing their health status, reflecting various physiological and metabolic processes within the organism [36]. Increased activities of AKP and ACP are often linked with various physiological and pathological conditions and can indicate tissue damage, inflammation, and changes in environmental stress levels [37]. Research has shown that when Mozambique tilapia (*Oreochromis mossambicus*) is exposed to the organic pesticides MCP and RPR-V, an increase in AKP and ACP enzyme activities in their serum is observed [38,39]. AST, a marker for hepatocellular damage, can decrease activity due to chemical stimulation, suggesting impaired liver function and hepatocyte death. Studies also indicate that when Nile tilapia (*Oreochromis niloticus*) is exposed to a Cypermethrin concentration of 0.05 μg·L^−1^, a decrease in serum AST activity and hepatocyte necrosis occurs [40]. Similarly, research by TL Kharat et al. on freshwater fish *Rasbora daniconius* exposed to 41% glyphosate found a reduction in serum AST activity and hepatocyte necrosis under these conditions [41]. In the current study involving carbaryl exposure, peak serum levels of AKP and ACP in juvenile *L. calcarifer* were observed at 24 h, possibly reflecting the most intense stress response to carbaryl exposure at this time point, leading to increased expression of these enzymes. Moreover, the trend of decreasing AST activity with prolonged carbaryl exposure observed in this study aligns with the literature, suggesting that carbaryl exposure may impair liver function and lead to hepatocyte necrosis. Relative to the trends observed within the control group, the activity of AKP at 72 h was significantly lower than at other time points. This may be due to cells being in an active phase of division and proliferation during the early stages of the experiment, maintaining higher AKP activity. As the cells progress into later stages, changes in the cell cycle or a slowdown in cellular proliferation could lead to a decrease in enzyme activity. Additionally, the significant reduction in ACP activity observed at 24 h, compared to 12 h and 72 h, may be attributed to the metabolic regulation mechanisms of liver cells, which can cause variations in enzyme activity at different time points. At 24 h, adjustments in metabolic pathways or intrinsic regulatory mechanisms of the enzyme, such as negative feedback inhibition, could result in decreased ACP activity.

Malondialdehyde (MDA) levels are commonly used to assess the oxidative stress state within organisms. Oxidative stress arises when the balance between free radicals (primarily oxygen radicals) and antioxidants is disrupted [42]. Elevated levels of MDA indicate that lipid peroxidation and cellular component damage are occurring, which could negatively impact cell function [43]. Research has shown that exposure to heavy metals and pesticides can increase MDA levels in fish [44]. However, in this study, there were no significant differences in the MDA content in the serum of juvenile *L. calcarifer* exposed to carbaryl compared to the control group, suggesting that the fish might be compensating for the increase in MDA through their intrinsic antioxidant mechanisms, thereby maintaining a relatively stable oxidative state.

Creatinine is a metabolic byproduct of muscle tissue and is also excreted by the kidneys. When renal function is impaired, creatinine accumulates in the body. Clinically, measuring serum creatinine is a common method for diagnosing kidney diseases [45]. In this comprehensive study, we meticulously tracked the creatinine levels in juvenile *L. calcarifer* as exposure time to carbaryl increased. Our findings revealed that these levels were significantly elevated in the experimental group compared to the control group at the 24 h, 48 h, and 72 h marks, with the highest concentration observed at 48 h. This pronounced peak in creatinine levels strongly suggests a potential impairment of renal function, indicating that carbaryl exposure may be deleterious to the kidney health of these juvenile fish. The consistent elevation across these time points underscores the severity and persistence of carbaryl’s impact on renal integrity, highlighting the need for further investigation into its chronic effects on aquatic life.

### 4.3. Effects of Chemical Pesticide Exposure on Organs and Tissues

Fish tissue histology is a crucial research tool in aquacultural science and environmental biology, enabling the identification of infections, tumors, nuclear deformations, and signs of cellular necrosis and other noninfectious diseases through the examination of various tissues such as the liver, kidneys, gills, and spleen [46]. Studies have shown that the liver is vital for processing and eliminating toxins from the body and is susceptible to cellular edema and necrosis under pesticide exposure and heavy metal contamination [47]. The head kidney and spleen are major immune organs in fish, and their melanomacrophage centers (MMCs) increase in number in response to chemical pollutants [48]. This is because MMCs can rapidly repair head kidney and spleen cells when they undergo necrosis [49]. In this study, we closely examined the effects of prolonged exposure to carbaryl on juvenile *L. calcarifer*. As the duration of exposure increased, distinct pathological changes were noted in the liver, including nuclear shrinkage, edema, and necrosis. These alterations indicate significant cellular stress and damage within hepatic tissues. In addition to these liver changes, we observed a marked increase in melanomacrophages (MMCs) in both the head kidney and spleen, suggesting a systemic response to the toxic exposure. These findings clearly demonstrate that carbaryl, a widely used chemical pesticide, not only affects the liver but also has a profound impact on multiple organ systems, leading to cellular edema, nuclear shrinkage, and necrosis in the liver, as well as immunological responses in the head kidney and spleen. The presence of MMCs, which are involved in the immune response and the clearance of damaged cells, further underscores the broad toxicological implications of carbaryl exposure in aquatic species. This comprehensive examination highlights the critical need for evaluating the environmental and health risks associated with carbaryl usage, particularly in aquatic environments where juvenile fish are exposed.

### 4.4. Changes in the Expression of Immune Genes in Fish by Chemical Pesticides

HSP90 (Heat Shock Protein 90) and HSP70 (Heat Shock Protein 70) are primary molecular chaperones in cells, playing critical roles in aiding protein folding, preventing misfolding and aggregation, facilitating the degradation of damaged proteins, and regulating cellular signal transduction [50]. During cellular stress from heat shock or chemical agents, accumulated unfolded proteins trigger the trimerization of heat shock factor (HSF) and its migration to the nucleus to activate heat shock elements (HSEs), initiating the transcription of heat shock protein genes such as HSP70 and HSP90 [51]. Chemical toxins, heavy metals, and other environmental pollutants can increase the quantity of unfolded or misfolded proteins, thereby stimulating an increase in the expression of HSP90 and HSP70 [52]. However, some studies have shown that specific pesticides can suppress the expression of the HSP70 transcription factor [53]. Da Rosa et al. observed in their study that when zebrafish (*Danio rerio*) were exposed to the chemical pesticide MPBI for 96 h, there was a downregulation in the expression of the HSP70 gene in their brains [54]. In this study, the expression levels of the HSP90 gene in the experimental group increased with prolonged exposure to carbaryl, suggesting that continuous exposure to the pesticide carbaryl may lead to an increase in the number of improperly folded proteins in the cells of L. calcarifer juveniles, thereby enhancing the expression of HSP90. Conversely, the expression of HSP70 showed a declining trend with increased exposure time. This could be due to carbaryl’s interference with the normal signaling pathways that promote HSP70 expression, possibly by competitively binding to transcription factors or altering their activity, thus inhibiting the synthesis of HSP70 mRNA.

The C3 protein, encoded by the complement C3 gene, is a central component of the complement system, and its activation is crucial for inflammatory responses and pathogen clearance mechanisms. In fish, immune cells such as macrophages and neutrophils produce cytokines in response to pathogen infection or environmental changes, which can activate the transcription of the C3 gene [55]. Previous studies have shown that in carp tissues, the expression of the C3 gene increases under exposure to 75 µg/L of the pesticide diazinon [56]. In our study, the expression levels of the C3 gene in the experimental group were only slightly higher than those in the control group at 48 h and exhibited a minor increasing trend, while at other times, they were lower than those in the control group, which is somewhat inconsistent with the findings mentioned above. This suggests that exposure to the chemical pesticide carbaryl for 48 h may slightly enhance the expression of the C3 gene in juvenile *L. calcarifer* due to environmental stress.

The IL-8 gene encodes an important proinflammatory cytokine, primarily secreted by monocytes, macrophages, epithelial cells, and hepatocytes during inflammatory responses [57,58]. Chemical pesticides may lead to the overactivation of transcription factors such as NF-κB and AP-1, which are directly involved in the upregulation of the IL-8 gene [59]. Eman Zahran and colleagues conducted a study on the gene expression levels in Nile tilapia (*Oreochromis niloticus*) exposed to the pesticide diazinon, finding a significant increase in the IL-8 gene among immune cells within one day of exposure to a concentration of 15 µg/L [60]. In our study, there was an upregulation of the IL-8 gene expression at 24 h under exposure to carbaryl, which could be attributed to inflammatory responses in the organ tissues of juvenile *L. calcarifer* induced by carbaryl stress, leading to an increase in IL-8 gene expression in immune cells.

## 5. Conclusions

In summary, this study investigated the effects of carbaryl exposure on juvenile *L. calcarifer* by setting experimental conditions at 0.5 ppm for 12, 24, 48, and 72 h. The study assessed various parameters including hepatic antioxidant enzyme activity, serum biochemical indices, histopathological changes in tissue organs, and immune gene expression levels, all of which were affected to varying degrees. Notably, as the duration of carbaryl exposure increased, a decline in hepatic antioxidant enzyme activity and changes in serum biochemical indices and immune gene expression levels were observed. These alterations suggest that exposure to carbaryl could potentially damage liver, kidney, and spleen functions in fish, and might also enhance the expression of certain immune genes. It was observed that after exposure to 0.5 ppm of carbaryl for 72 h, the activities of CAT and GSH-PX in the liver of juvenile *L. calcarifer* reached their lowest levels, with significant changes starting at 12 h. Additionally, the highest levels of creatinine were recorded after 48 h of exposure to 0.5 ppm of carbaryl, with significant changes starting at 24 h. This research not only provides fundamental data for evaluating the biochemical and immunological impacts of carbaryl toxicity on fish but also lays the groundwork for further studies on protective strategies against pesticides in aquaculture.

## Figures and Tables

**Table 1:** Primer of the immune-related genes in *L. calcarifer* used in qPCR.

Gene Abbreviation	Primer Sequence (5′–3′)	Amplicon Size (bp)	Accession No.
β-actin	F: AACCAAACGCCCAACAACT	112	XM_018667666
	R: ATAACTGAAGCCATGCCAATG		
HSP90	F: ACGATGATGAGCAGTATGCC R: CAAACAGGGTGATGGGGTA	201	XM018661637
HSP70	F: CTGGAGTCCTACGCTTTCAA R: CTTGCTGATGATGGGGTTAC	204	HQ646109
C3	F: AAATGCTGCCATCGTTCC	175	XM_018679796
	R: CCAGTGACCTTCAGACCAAA		
IL-8	F: TCTGACTGTTCCTGAGGCTATC R: GACGTCCAATGGGCTTTCT	92	XM_018695863

## Data Availability

The data that support the findings of this study are available from the corresponding author upon reasonable request.

## Supplementary Materials

The following supporting information can be downloaded at: https://www.mdpi.com/article/10.3390/jox14030051/s1, Table S1. Analysis of the significant differences in CAT enzyme activity changes in the liver of juvenile *L. Calcarifer* due to the interaction between carbaryl exposure and duration of exposure. Table S2. Analysis of the significant differences in LDH enzyme activity changes in the liver of juvenile *L. Calcarifer* due to the interaction between carbaryl exposure and duration of exposure. Table S3. Analysis of the significant differences in GSH-PX enzyme activity changes in the liver of juvenile *L. Calcarifer* due to the interaction between carbaryl exposure and duration of exposure. Table S4. Analysis of the significant differences in SOD enzyme activity changes in the liver of juvenile *L. Calcarifer* due to the interaction between carbaryl exposure and duration of exposure. Table S5. Analysis of the significant differences in serum AKP levels of juvenile L. Calcarifer due to the interaction between carbaryl exposure and duration of exposure. Table S6. Analysis of the significant differences in serum ACP levels of juvenile *L. Calcarifer* due to the interaction between carbaryl exposure and duration of exposure. Table S7. Analysis of the significant differences in serum MDA levels of juvenile *L. Calcarifer* due to the interaction between carbaryl exposure and duration of exposure. Table S8. Analysis of the significant differences in serum AST levels of juvenile *L. Calcarifer* due to the interaction between carbaryl exposure and duration of exposure. Table S9. Analysis of the significant differences in serum creatinine levels of juvenile *L. Calcarifer* due to the interaction between carbaryl exposure and duration of exposure.
