# Supplementary material for: Toxic Effects of Carbaryl Exposure on Juvenile Asian Seabass (Lates calcarifer)
Source: J Xenobiot. 2024 Jul 10;14(3):923–38. doi: 10.3390/jox14030051 (PMC11270272; doi:10.3390/jox14030051)
Supplement: Supplementary file 1 [file jox-14-00051-s001.zip › jox-3038893-supplementary.pdf]

Table S1: Analysis of the significant differences in CAT enzyme activity changes in the liver of juvenile *L. Calcarifer* due to the interaction between carbaryl exposure and duration of exposure.

Source	Sum of Squares	Degrees of freedom	F-Value	P-Value
Corrected Model	2873.35	7	237.70	
Group	1679.02	1	972.40	0.001
Time	1103.93	3	213.11	0.001
Group*Time	90.40	3	17.45	0.001
Error	27.63	16		

Table S2: Analysis of the significant differences in LDH enzyme activity changes in the liver of juvenile *L. Calcarifer* due to the interaction between carbaryl exposure and duration of exposure.

Source	Sum of Squares	Degrees of freedom	F-Value	P-Value
Corrected Model	270298.88	7	14.64	
Group	146684.44	1	55.61	0.001
Time	120921.25	3	15.28	0.001
Group*Time	2693.19	3	0.34	0.796
Error	42202.66	16		

Table S3: Analysis of the significant differences in GSH-PX enzyme activity changes in the liver of juvenile *L. Calcarifer* due to the interaction between carbaryl exposure and duration of exposure.

Source	Sum of Squares	Degrees of freedom	F-Value	P-Value
Corrected Model	268.98	7	6.99	
Group	213.49	1	38.85	0.001
Time	29.77	3	1.81	0.187
Group*Time	25.27	3	1.56	0.238
Error	87.91	16		

Table S4: Analysis of the significant differences in SOD enzyme activity changes in the liver of juvenile *L. Calcarifer* due to the interaction between carbaryl exposure and duration of exposure.

Source	Sum of Squares	Degrees of freedom	F-Value	P-Value
Corrected Model	85.87	7	1.05	
Group	2.73	1	0.23	0.635
Time	79.80	3	2.28	0.118
Group*Time	3.35	3	0.1	0.961
Error	87.91	16		

Table S5: Analysis of the significant differences in serum AKP levels of juvenile L. Calcarifer due to the interaction between carbaryl exposure and duration of exposure.

Source	Sum of Squares	Degrees of freedom	F-Value	P-Value
Corrected Model	126.69	7	5.19	
Group	16.83	1	4.82	0.043
Time	42.64	3	4.07	0.025
Group*Time	67.21	3	6.42	0.005
Error	55.83	16		

Table S6: Analysis of the significant differences in serum ACP levels of juvenile *L. Calcarifer* due to the interaction between carbaryl exposure and duration of exposure.

Source	Sum of Squares	Degrees of freedom	F-Value	P-Value
Corrected Model	676.09	7	4.70	
Group	266.13	1	12.96	0.002
Time	70.27	3	1.14	0.363
Group*Time	339.68	3	5.51	0.009
Error	328.58	16		

Table S7: Analysis of the significant differences in serum MDA levels of juvenile *L. Calcarifer* due to the interaction between carbaryl exposure and duration of exposure.

Source	Sum of Squares	Degrees of freedom	F-Value	P-Value
Corrected Model	101.54	7	1.41	
Group	0.93	1	0.09	0.768
Time	95.35	3	3.08	0.057
Group*Time	5.25	3	0.17	0.915
Error	164.99	16		

Table S8: Analysis of the significant differences in serum AST levels of juvenile *L. Calcarifer* due to the interaction between carbaryl exposure and duration of exposure.

Source	Sum of Squares	Degrees of freedom	F-Value	P-Value
Corrected Model	289.16	7	8.36	
Group	117.48	1	23.79	0.001
Time	125.95	3	8.5	0.001
Group*Time	45.73	3	3.09	0.057
Error	146.08	16		

Table S9: Analysis of the significant differences in serum creatinine levels of juvenile *L. Calcarifer* due to the interaction between carbaryl exposure and duration of exposure.

Source	Sum of Squares	Degrees of freedom	F-Value	P-Value
Corrected Model	6394.30	7	100.05	
Group	3056.88	1	334.82	0.001
Time	1714.38	3	62.59	0.001
Group*Time	1623.04	3	59.26	0.001
Error	146.08	16		
